# The digital workplace and meeting accessibility: A qualitative study on listening effort in video meetings for employees with hearing loss

**DOI:** 10.1177/10519815251398498

**Published:** 2025-12-08

**Authors:** Erik Marsja, Carine Signoret, Victoria Stenbäck

**Affiliations:** 1 Disability Research Division, Department of Behavioural Sciences and Learning, Linköping University, Linköping, Sweden; 2 Division of Education, Teaching and Learning, Department of Behavioural Sciences and Learning, Linköping University, Linköping, Sweden

**Keywords:** remote work, mental fatigue, videoconferencing, communication barriers, hearing aids, cochlear implants, telework, persons with hearing disabilities

## Abstract

**Background:**

Hearing loss is a common workplace disability that presents communication challenges. While digital communication platforms can offer opportunities for flexibility and inclusion, they may also present barriers for individuals with hearing loss. More research is needed to understand how video meetings are experienced by these employees and how to create more inclusive and effective work environments.

**Objective:**

This study aimed to explore the positive and negative aspects of video meetings for employees with hearing loss and identify strategies they use to enhance communication.

**Methods:**

We conducted a qualitative study using an abductive approach to thematic analysis. Fourteen employees with hearing loss, aged 35 to 67 years, were interviewed. All participants used hearing aids except for one who had a cochlear implant. They were recruited through professional networks and audiology clinics.

**Results:**

Listening effort emerged as the overarching theme, experienced as both physically and mentally demanding. Sub-themes included both positive and negative experiences, the effects on participation, and strategies for managing communication challenges. Participants emphasized the importance of high-quality audio, video, captioning, and structured turn-taking. Workplace support, technical solutions, and remote work opportunities were described as helpful in reducing listening effort and improving inclusion.

**Conclusions:**

Employees with hearing loss report both benefits and challenges in video meetings. Based on participants’ experiences, workplace measures such as captioning, high-quality audio, and inclusive communication practices may support participation. Allowing remote work and structuring hybrid meetings with clear turn-taking rules may support well-being and participation. Future research should assess the effectiveness of these approaches.

## Introduction

Hearing loss is a widespread chronic condition affecting approximately 1.57 billion people worldwide, or one in five individuals.^1^ Of these, over 60% are aged 50 or older, indicating that many affected individuals are of working age. A recent systematic review of nearly 100 studies conducted across 40 countries reported a global adult prevalence of 31%, with considerable variation across regions, age groups, and definitions of hearing loss.^2^ As digital communication becomes increasingly central to modern work,^3^ examining how employees experience these changes with hearing loss is essential. This study aims to explore employees’ experiences with hearing loss during video meetings, focusing on both the positive and negative aspects of these interactions.

Employees with hearing loss face unique challenges in the workplace, especially in spoken communication. For many individuals, particularly hearing aid users, difficulties arise in meetings, phone calls, and noisy environments.^4^ These challenges require extra listening effort, leading to fatigue, irritability, and difficulty focusing.^5^ The harder the task, the more mental energy it demands, which often results in greater fatigue than in persons with typical hearing.^6–8^ These issues can negatively impact physical and mental well-being,^9,10^ hinder performance^11^ and participation.^4^ Employees may feel less in control during meetings, participate less, or mentally disengage,^12,13^ or experience frustration when talking with coworkers.^14^

Increased listening effort can impinge upon life outside of work. Effort-related fatigue has been linked to poorer health and quality of life,^15^ such as increased risk of cardiovascular diseases, depression, or insomnia.^16,17^ It also contributes to a greater need for recovery,^18,19^ with employees with hearing loss often requiring more extended recuperation than peers with normal hearing.^8,11^ Many also report feeling too exhausted for social activities after work,^20^ especially when exposed to noisy environments.^21^ Poor audio and visual conditions in video meetings can worsen communication challenges and lead to long-term social and work disadvantages.

While technology has advanced, offering various solutions for hearing loss, such as improved hearing aids, these advancements alone cannot address all workplace challenges.^22^ For example, in Sweden and South Korea, hearing aids are subsidised and commonly used,^23,24^ yet additional adjustments are needed to ensure an inclusive and supportive work environment.^25^ Despite improvements in communication, hearing aids have limitations, particularly in noisy environments or group settings,^26^ as well as when background noise and multiple speakers are present.^27^ Thus, good communication strategies and workplace adaptations are necessary to supplement individual and contextual factors.

To understand the broader range of factors contributing to hearing-related challenges, the Framework for Understanding Effortful Listening (FUEL) proposes that various factors influence listening effort and provides a comprehensive, multidimensional framework for understanding these effects and their outcomes.^5^ According to FUEL, hearing loss can increase listening effort by reducing the listener's auditory input, requiring more cognitive resources to compensate for the degraded auditory signal. Video meetings can pose additional challenges for employees with hearing loss, such as poor sound quality, lack of visual cues, or increased multitasking. These factors could further increase listening effort and negatively affect attention, comprehension, and memory. The FUEL consists of five main factors influencing perception of a spoken message: the source, the transmission, the listener, the message, and the context. First, the source factor is related to the talker's characteristics and includes the accent or the emotion of a talker. Second, the transmission factor refers to the acoustic characteristics of speech, background noise, and visual cues that can enhance or hinder speech comprehension. Third, the listener factor includes the individual's auditory (e.g., hearing ability), cognitive, motivational skills, and personal and situational factors such as age or fatigue. Fourth, the message factor involves the cognitive and linguistic demands of the situation, such as the speech's complexity, speed, or familiarity. Finally, the context factor encompasses both the physical and social environments, including the situational settings or social expectations. Listening effort arises from interacting with these factors and their alignment with the listener's communicative goals. In the present study, it is expected that these challenges cause greater fatigue, stress, and dissatisfaction in video meetings for individuals with hearing loss compared to those with typical hearing.

Numerous factors influence listening effort, including human, technological and environmental factors. Human factors include hearing ability, cognitive capacity,^28,29^ and attentional strategies.^30^ For instance, individuals with hearing loss often rely on visual cues such as speechreading and facial expressions to support speech comprehension and increase communication quality. Still, these strategies demand additional cognitive resources.^31^ Technological and environmental factors include degraded auditory and visual signals, such as poor sound quality or transmission delays, which impede the listener's ability to comprehend speech. For example, backlighting, where a light source behind the speaker obscures their facial features and lip movements, can affect visual strategies like speechreading.^31^ Moreover, visual clutter from a messy or busy background, pop-up notifications, or other windows on the screen can divert the listener's attention^32^ and increase cognitive load.^33,34^ Other examples include acoustic challenges such as background noise, the number of target speakers, or overlapping conversations. Competing noise sources, whether from other people or environmental sounds like traffic, can mask the speaker's voice, reduce speech intelligibility and increase cognitive load.^35^ These human, technological, and environmental factors often overlap in video meetings, compounding the listening challenges.

Research on the impact of video meetings and hearing loss is limited, but some studies have touched upon this topic. For example, Pinsonnault-Skvarenina et al.^36^ conducted a quantitative survey study during the COVID-19 pandemic, reporting that individuals with hearing loss faced communication challenges and psychosocial impacts, including in video meetings. Chauvette et al.^37^ carried out a qualitative interview and found mixed experiences with videoconferencing, with benefits including visual cues and quieter settings, while drawbacks involved poor audio/video quality, background noise, and technical issues. Both studies focused primarily on pandemic-related changes rather than video meeting experiences per se.

The current study explored the positive and negative aspects of video meeting tools on listening effort and participation for employees with hearing loss. As discussed in the introduction, video meetings involve various factors that can degrade the sound and visual quality of the communication, such as network bandwidth, audio compression, microphone and speaker quality, lighting, and lip synchrony.^35^ Given the lack of research in this area, the present study can generate hypotheses for future research. It can also inform workplace policies and technological adaptations to better support these employees, ensuring a more inclusive and effective work environment.

## Method

This study adopted a qualitative design and received ethical approval from the Swedish Ethics Authority (DNR: 2022-01119-01).

### Participants

Fourteen employees with hearing loss (five men, nine women), aged 35–67 (*M* = 53 years), were interviewed. All were native Swedish speakers and used hearing aids except for one who had a cochlear implant, with self-reported hearing loss ranging from mild to profound (see Table 1). They were recruited through an invitation email sent to an extensive mailing list, the Swedish Hearing Health Registry, which included people working with hearing loss (e.g., audiologists) and individuals with hearing loss. Recruitment also took place through posters on bulletin boards at various audiological clinics in Sweden. After expressing interest by emailing the responsible researcher, potential participants received detailed information about the study via an information letter (in an attached PDF). After reading the letter, interested participants emailed the main author to schedule an interview.

**Table 1.** Participants’ level of hearing loss, occupation, and auditory assistive devices used.

Participant	Level of hearing loss	Occupation	Auditory assistive devices
Male participant 1	Profound	Assistive Technology Specialist	Cochlear Implants, External Microphone
Male participant 2	Mild	IT Engineer	Hearing Aids, External Microphone
Male participant 3	Moderate	Engineer	Hearing Aids
Male participant 4	Mild	Priest	Hearing Aids, External Microphone
Male participant 5	Moderate	Researcher	Hearing Aids
Female participant 1	Moderate	University Administrator	Hearing Aids, External Microphone
Female participant 2	Severe	Advisor	Hearing Aids, External Microphone
Female participant 3	Moderate	Audiologist	Hearing Aids
Female participant 4	Mild	Special Education Teacher	Hearing Aids
Female participant 5	Mild	Advisor	Hearing Aids
Female participant 6	Moderate	Special Education Teacher	Hearing Aids
Female participant 7	Severe	Sign Language Teacher	Hearing Aids
Female participant 8	Mild	Parish Educator	Hearing Aids
Female participant 9	Mild	Teacher	Hearing Aids

*Note.* External microphones include different types of Bluetooth devices that can be connected to hearing aids.

The sample size was guided by information power, indicating that narrow aims and homogenous, rich data need fewer participants..^38^ In this study, combining interviews with a relatively homogenous group led to sufficient information power with fourteen participants (see also 2.4 Thematic analysis). Information power, unlike statistical power, suits qualitative research focused on depth over generalizability.^39^ We evaluated sample adequacy during data collection and found that interviews conducted after the first ten reinforced existing themes rather than introducing new ones.

### Interviews

The interviews were all conducted via Microsoft Teams by the first author. Beforehand, the participants signed a digital consent form. After consenting, they answered demographic questions on age, self-reported hearing loss, and use of hearing aids or cochlear implants. The semi-structured interviews followed an interview guide, including questions about participants’ perspectives on video meetings, the challenges and benefits of remote work, and comparisons between digital and face-to-face interactions. The FUEL framework^5^ was used to inform the development of the interview guide and support early deductive coding; it was not applied as a fixed analytical structure, but ensured that key aspects of listening effort were included, including listener abilities, message complexity, transmission quality, and context. Moreover, the interview guide was developed using a deductive approach, meaning that the questions reflected key topics of interest (challenges and advantages of video meetings) based on theoretical assumptions and previous research.^39^ This ensured that key domains of interest were addressed while allowing space for unanticipated insights.

The interview guide aimed to encourage participants to reflect on their experiences in video meetings, both positive and negative, and to share their thoughts about the challenges and advantages of remote work. The guide did not limit participants to specific scenarios and allowed them to discuss a variety of video meeting contexts. Participants were encouraged to reflect on situations where digital tools facilitated or hindered participation. This flexible yet structured format made semi-structured interviews appropriate for capturing the detailed and context-specific nature of participants’ experiences. To ensure a comprehensive understanding, participants were also asked to reflect on how their hearing loss affected their work life and how their experiences with video meetings differed from those with face-to-face meetings, especially regarding physical and mental engagement. The interview guide is provided as Supplementary Material.

Each interview lasted approximately 40 to 90 min, depending on the amount of information the participants had to share and the depth of their responses. To ensure confidentiality, all interviews were audio-recorded with participants’ consent and transcribed verbatim by the first author. Transcriptions were pseudonymised to remove any identifiable information. Efforts were made to create a comfortable and respectful environment (e.g., by ensuring clear sound and video), allowing participants to freely express their views and opt out of questions or the interview entirely at any point without explanation.

### Thematic analysis

Thematic analysis was selected for its flexibility and suitability for identifying patterns across participants’ experiences and perspectives. We used an abductive^40^ approach to thematic analysis to identify and analyse themes.^39,41^ The abductive approach mixes deductive and inductive reasoning, using prior knowledge while avoiding absolute truths.^40^ The analysis aligned with what Braun and Clarke^39^ describe as coding reliability, moving towards codebook thematic analysis. Coding reliability ensures accuracy, treating themes as topic summaries, while codebook thematic analysis extends deductive approaches by allowing exploratory analysis. A coding reliability and codebook thematic analysis approach allowed for new knowledge and the formation of themes beyond theoretical assumptions and previous research, hence, more abductive reasoning. The coding reliability approach allowed for themes to form based on topic summaries in the data, reflecting questions from the interviews. Using codebook thematic analysis allowed the analysis to develop further and guide continuous data coding beyond deductive assumptions.

Data were analysed using NVivo 12,^42^ enabling systematic coding and thematic analysis to identify key patterns and relationships within the interview data. The main topics from the interview guide allowed nodes to be created from the data, containing references describing the topics. Nonetheless, the material was read through multiple times by two of the researchers (EM & VS) to find new codes or subcodes not previously thought of as predetermined topics before forming themes. To enhance credibility, two researchers independently coded transcripts and resolved differences through discussion. The abductive approach kept the analysis grounded in participants’ accounts while allowing themes to emerge beyond the FUEL, enabling both theory-informed and data-driven interpretations.

## Results

The results section presents the thematic analysis, main theme, subthemes, quotes, and a synthesis of key video meeting advantages and challenges.

### Listening effort

Much of the focus in the interviews revolved around listening effort and how it influenced the participants as individuals, both physically and mentally. Listening effort was the overarching theme, and most subsequent emerging themes were related to listening effort. The most prominent themes related to listening effort found during the analyses were *Positive experiences of video meetings*, *Negative experiences of video meetings*, how *Participation* is affected positively and negatively, and different *Strategies* used by employees with hearing loss in work situations. For an overview of these themes and subthemes, see Table 2.

**Table 2.** Themes and subthemes identified through thematic analysis.

Main theme	Theme	Subtheme
Listening effort	Positive aspects of video meetings	Better hearing possibilities
		Turn-taking
		Accessibility
		Equal Terms
	Negative aspects of video meetings	Digitally tired
		Mental fatigue
		Large meetings
		Technological aspects
		Hybrid Meetings
	Participation	
	Strategies	My own responsibility
		Distribute workload
		Take control.
	Support	Leadership
		Workplace support
		Recovery

*Note.* The theme Participation did not include subthemes.

#### Positive aspects of video meetings

Some participants described positive experiences with video meetings, highlighting themes such as *better hearing possibilities*, clearer *turn-taking*, increased *accessibility*, and a sense of participating on *equal terms*. These benefits were especially noted when platforms allowed structured turn-taking or participants could join from quieter environments. While not universal, such experiences point to specific conditions under which video meetings can support inclusion for employees with hearing loss.

##### Better hearing possibilities

One of the subthemes that emerged was better hearing possibilities. The participants also emphasised better turn-taking, accessibility, and meetings on more equal terms. One participant noted that she hears much better in a digital meeting:

[…] it's wonderful. I can hear what people say now and I don’t have to guess what people are saying or ask questions all the time. (Female Participant 3, aged 65, moderate hearing loss).

Another participant said:

It has the positive effect that the sound comes directly in […]. There is not so much noise around […] it's easy to concentrate. I also feel that thanks to digitalisation, […] fewer meetings […] don’t have these long, tiring meetings. So that's a positive effect as well. I think […] in video meetings or when it's digital, there is a clearer agenda. There is not much going on outside the topic of the meeting, […], that makes it easier for me to keep up. (Male participant 5, aged 51, moderate hearing loss).

##### Turn-taking

Turn-taking was described as important to meetings and interactions. Without working turn-taking, participants find it challenging to know when to join the conversation, and irritation can occur. One participant said:

So my co-workers don’t understand this. For them, it's kind of just to come in when it gets quiet. That's not the case for me […] because it takes a while before it gets quiet. During morning meetings, I can get angry that I never get to speak. But then we have a workplace meeting and then we have this hand-raising function in Teams, […], that you raise your hand. And […] it works quite well. (Male participant 1, aged 49, profound hearing loss).

Another participant said:

At least it has the advantage, I think, that people talk a little more one by one. It won’t be so chatty. That's a big difference, I think. […]. So, what can you say? […] I still think it's much easier for people to talk one by one, I’ve realised. (Female participant 1, aged 52, moderate hearing loss).

##### Accessibility

Another positive aspect highlighted by the participants was increased accessibility, such as not having to travel to meetings or face loud and tiring environments to and from work. One participant said:

This digitalisation meant that you didn’t have to go to all these meetings […] you can sit in a different environment and have meetings instead.

There are also very, very many meetings now and then. I can sit in the car and have meetings. I can sit anywhere, that is. I don’t get physically tied to a certain place […], my work situation becomes much more flexible and makes me work more efficiently. So, digital meetings, I would like to have them all the time. (Male participant 3, aged 65, moderate hearing loss).

Other participants thought digital meetings decreased the stress of having to travel by car and face possible noisy environments to and from a meeting:

We have had long journeys both there and home, humming cars, trains, buses, or whatever it is that has taken energy before coming to the meeting. I don’t have to do this in the digital meeting, especially at home, so it's calm before [before the meeting]. […] there are many things around a physical meeting that can be tiring with hearing loss, travel, for example. (Female participant 5, aged 51, moderate hearing loss).

##### Equal terms

Several participants also highlight a level playing field, meetings on more equal terms. For example, one participant said:

There could be a little more equality in conditions, perhaps that a digital meeting requires more discipline and that everyone has to sharpen.

But I also had experiences of completely digital meetings, where I, for once, had the same conditions as everyone else. It was actually more on everyone's terms […] (Female participant 1, aged 52, moderate hearing loss).

#### Negative aspects of video meetings

Several participants described challenges associated with video meetings, particularly when technical or environmental conditions were suboptimal. Negative aspects included being *digitally tired,* having *mental fatigue, large meetings,* the *lack of proper technological aids,* and *hybrid meetings*.

##### Digitally tired

Some participants reported feeling more tired after digital meetings than after physical meetings. They described a general sense of being drained but were often unable to attribute this fatigue to a specific cause:

You get more tired from a digital meeting, strangely enough.[…] It [the meeting] drains you somehow anyway to meet digitally. […] it is doing something to us for sure. (Female participant 4, aged 49, mild hearing loss).

But many times, it can be so much disturbing noise or that some meeting tools or there has been a lot of humming from microphones and things like that when someone turns on and all that is extremely tiring. I get, I can say that I get tired 3 times as fast in my head because of this. (Male participant 2, aged 58, mild hearing loss).

##### Mental fatigue

Many participants attributed the reported tiredness to a negative impact on their mental well-being. They mentioned feeling mentally exhausted after digital meetings, with effects on memory and increased feelings of sadness. One participant said:

Yes, then I get tired and I notice that I don’t remember as well. […] There was some screeching sound and like and sometimes I don’t have the energy to say it. And the consequence I notice after such a meeting is that I have not remembered as much as they said as it sounds bad. (Female participant 5, aged 53, mild hearing loss).

Another participant said:

And if you’re going to walk around with a mic, then you should also wait. And then you have to give it [the microphone] before the person starts talking. Then everyone is involved, and everyone has access to people who speak. It's very important and can make me sad that people don’t understand it. Then I can get really tired after that meeting, and sad and a little vulnerable. (Female participant 6, aged 67, moderate hearing loss).

##### Large meetings

Meetings with many participants were experienced as something difficult to handle. This was due to a lack of structure, some having their cameras off, difficulty seeing the faces of meeting participants, and poor turn-taking. For example, one participant said:

If there were too many, I can’t decide who is who either. It will be too hard. (Female participant 3, aged 40, moderate hearing loss).

One participant said:

I’ve been to slightly larger meetings (40–50 people) and there I’ve noticed that people haven’t raised their hands, especially when you have digital meetings and don’t have the video but only the audio. Which makes it really hard to hear. (Male participant 1, aged 49, profound hearing loss).

##### Technological aspects

Meeting arrangement and participant equipment were experienced as something that could complicate the listening situation. One participant said:

Yes, it is extremely stressful as an individual with hearing loss when you end up in a meeting, when there is a poor connection, when the image becomes coarse-grained because they do not have a good enough camera. It can be a poor broadband connection […] It sways when they don’t have a good sound system, and you can’t hear. Some people use in-ear headphones, and the sound is much worse. (Female participant 9, aged 52, mild hearing loss).

Another example was:

And it's just as important in digital meetings that you sit in a room with good acoustics. It makes it easier when you have a headset and the microphone is close to your mouth, and so on. (Female participant 2, aged 52, mild hearing loss).

Then there was someone who was going to talk […] who had bad sound, who had such a hands-free cord with a mic on the cord, and it was a bit of an echo. She was quite unclear in her voice, just mumbling like that. (Female participant 4, aged 49, mild hearing loss).

##### Hybrid meetings

Another difficulty experienced by the participants was when meetings were in a hybrid format and people were present physically in a meeting room as well as digitally:

it's difficult with turn-taking […] they could be having their own conversation by the table and […] it creates a lot of frustration and fatigue because it's difficult to have a good conversation. […] No, I don’t like hybrid meetings. I think that no, I think this hits the mark. Those are probably the meetings that hit me the hardest when you have hearing loss actually and if you are the one participating digitally. (Female participant 5, aged 51, moderate hearing loss).

#### Participation

Participants experienced both benefits and challenges related to their participation in digital settings, spanning social interactions and meeting engagement. For instance, one participant said:

Most people want physical meetings to have that interaction […] the meeting dynamics to talk and laugh which is of course […] but for someone with hearing loss you’re not a part of that meeting. […] It's nice to be at work too and meet people, but at the same time it was very nice to be at home because you can eat by yourself in peace and quiet and you can get in touch with the people you want to talk to. (Female participant 1, aged 52, moderate hearing loss).

Another participant described:

So that in a way it is perhaps [it] calls for more participation in the digital space if not too many participants at the same time, but in up to maybe eight people somewhere, I would say that participation increases because it becomes so clear who has contributed or interacted or said something, so to speak. (Male participant 2, aged 52, mild hearing loss).

#### Strategies

To deal with some of the difficulties the participants experienced in digital meeting settings, they used a series of strategies to cope with various complications. The most prominent strategies emerging from the analysis were that the participants acknowledged that it was their *own responsibility* to make the work situation function, to *distribute the workload* wisely, and to *take control* of the situation.

##### My own responsibility

Some participants highlighted that to make the situation work, they found that the responsibility fell upon themselves. Here are some examples of the descriptions by two participants:

And that somewhere this whole responsibility […] what my work situation looks like has in many ways fallen on to me. (Female participant 8, aged 62, mild hearing loss).

But absolutely this is […] that it is possible to control yourself versus nothing happening, so to say that you have to ask the participant to raise [the volume] their microphone. (Male participant 2, aged 52, mild hearing loss).

##### Distribute workload

In the interviews, the participants discussed the importance of economising their energy by distributing the workload wisely. One participant said:

Then I have an opportunity to adjust that it's not like meetings back to back like this. I try to control it a bit, we are so autonomous here, so we can sort of regulate our own calendar like that. (Female participant 5, aged 51, moderate hearing loss).

Another described it as follows:

I made a priority and felt that, no, this is me deprioritising it versus the energy and power that it will take. (Female participant 2, aged 35, severe hearing loss).

##### Take control

Another strategy the participants used was taking control of the situation when they found it unmanageable. Some participants described the situation as follows.

But I have more power to influence in this video meeting because I can raise the volume. I see the person I have a greater impact, opportunities in the video meetings. So that I feel more involved in a video meeting. (Female participant 9, aged 52, mild hearing loss).

But I can say, I’m good at telling [others] when I can’t hear so that people repeat and so on. I think it works pretty well actually. (Female participant 5, aged 51, moderate hearing loss).

#### Support

Many participants emphasised the importance of support and understanding from colleagues and family in alleviating the strain caused by hearing impairment in the workplace or after a strenuous workday. Three themes emerged from this analysis: *Leadership*, *Workplace support*, and *Recovery*.

##### Leadership

Some participants highlighted the importance of supporting leadership for the work situation to be manageable. The participants emphasised what difference leadership can make:

For example, with our boss, have encouraged everyone in our department to use headsets with microphones for my sake actually, and everyone has been able to buy and usually use most […], but then it will be a very good meeting for me and I can relax. (Female participant 1, aged 52, moderate hearing loss).

I can say this because there is a lot in the video meeting. So I can feel that I have lost quite a lot of this social belonging. We’ve become a few pictures on the screen just for each other and it's something that we’re struggling with right now and it's tough I think because I can. I’m involved, but I can feel that I’m getting a little more isolated. […] I, I’m not excluded as our employer is trying to remind us all the time. (Female participant 9, aged 52, mild hearing loss).

##### Workplace support

Some participants also emphasised the importance of having understanding and helpful colleagues, as well as a viable work environment.

It has usually been the meetings that have been very good, and there have been, so to speak, people who have been aware that I have poor hearing. […] I think it is a positive image of sitting with people who may also have poor hearing. […] That you now have quite understanding work colleagues who work with the hearing, if I have to go to them, then I am quite straightforward when I say that I do not hear. (Male participant 1, aged 49, profound hearing loss).

I also have very good colleagues. I have one, my colleague who usually joins me, we often work together as she helps me along the way and she notices when I can’t hear, but, but it's tough. (Female participant 1, aged 52, moderate hearing loss).

##### Recovery

Many participants discussed the need for recovery, both physical and mental. Some highlighted that recovery was easier to achieve while working remotely from home.

I tell that I get tired faster, faster tired than the average [person]. This means that when I take a break, I work from home and work digitally. Then you take a certain break, which you sometimes call a micro-break. […] And I can when I work from home, you want to go out and get fresh air between 2 different activities. If you say so, I'll go out for a walk and then come back. It's difficult at work. (Male participant 5, aged 51, moderate hearing loss).

Here is an incredibly good sound environment. It's just my husband and me in the whole house. There is no one else who influences […] so I can control this and I appreciate that enormously. (Female participant 8, aged 62, mild hearing loss).

The thematic analysis identified both the benefits and challenges of digital meetings. Positives included clearer audio, better turn-taking, and greater accessibility. Many found it easier to hear, making participation less effortful. The structured turn-taking helped ensure equal engagement, while remote access reduced physical and environmental strain. Negatives included mental fatigue and difficulties in large or hybrid meetings. Many felt drained after video calls, struggling to focus and retain information. Large meetings were problematic due to poor turn-taking and a lack of structure, resulting in reduced participation. Hybrid meetings caused frustration and made following discussions more challenging, exacerbating feelings of isolation. To manage, participants adjusted their environment, managed their workloads, and utilised meeting controls to ease the effort.

## Discussion

This study aimed to understand employees’ experiences with hearing loss in video meetings using a qualitative, abductive approach. We explored their perceptions, strategies, and physical and psychological experiences. The study revealed five main findings. First, listening effort in video meetings was reported to depend on turn-taking, accessibility, hearing opportunities, technological aids, and meeting size. Second, video meetings affected social and professional participation: some felt included, while others experienced isolation. Third, participants used various coping strategies, including taking responsibility, managing workload, and maintaining control. Fourth, support from colleagues and leaders was crucial in reducing workplace strain. Finally, participants emphasised the need for physical and mental recovery, with remote work offering better recovery opportunities than office settings. It is also possible that cultural norms, organisational expectations, and job role demands shape individual experiences of listening effort and participation. These contextual influences may affect how employees perceive accessibility, cope with fatigue, or navigate disclosure of hearing loss.^4,43^

One important positive aspect of video meetings is identified in the subtheme *better hearing abilities.* Participants reported that they heard better in video meetings compared to other communication modes. This suggests that video meetings may overcome problems that previous research has found for individuals with hearing loss, such as communication difficulties during in-person meetings,^44^ telephone use, and the lack of suitable hearing aid technology in noisy environments.^4^ This aligns with the transmission factors of the FUEL,^5^ where a clearer auditory signal lessens the cognitive load required to process speech, thereby reducing listening effort and associated fatigue. Further explanations for this finding include reduced noise levels in optimal video meetings, direct sound transmission to headphones or hearing aids, and focusing on visual cues and facial expressions, allowing speechreading.^31^ Additionally, sound quality and clarity may improve when speakers use microphones, which will in turn ease speech comprehension.^45^ Specifically, our findings align with Hersh et al., who highlighted how video meetings benefited hearing-impaired users by improving lip-reading visibility, allowing volume adjustments for better clarity, and reducing overlapping voices or acoustic issues, making speech easier to follow.^46^ Another key finding within the positive aspects is the subtheme of *turn-taking*, which benefits employees with hearing loss. Turn-taking can reduce the noise that masks speech, thereby decreasing listening effort and enhancing speech understanding.^28,29^ Turn-taking occurs likely because even employees without hearing loss struggle to hear well without it. Within the FUEL,^5^ turn-taking could be interpreted as a message factor, as it decreases the complexity of the listening situation (i.e., by reducing the number of simultaneous talkers).

*Accessibility* was another positive subtheme, as video meetings allowed participation in work activities that would otherwise be difficult or impossible due to hearing loss. Our findings align with those of Hersh et al., who reported that video meetings facilitated participation for disabled workers, including those with hearing impairments, by reducing barriers associated with commuting and navigating in-person work environments.^46^ Participants in their study also perceived virtual meetings as less tiring due to the absence of travel demands. Some of our participants similarly valued avoiding travel and loud, exhausting settings, reinforcing the accessibility advantages of virtual communication. These aspects may contribute to lowering listening effort and increasing the well-being of employees with hearing loss. According to the context factors in the FUEL,^5^ it can be interpreted as enabling a more accessible environment where the participants experience better physical and social accessibility. While no previous studies have specifically reported this as a factor affecting listening effort, there is ample evidence that background noise can impair speech perception and increase cognitive load for individuals with hearing loss.^35^ Thus, video meetings may offer a more accessible and comfortable alternative, specifically when sound quality and visual cues are optimal. The subtheme of *equal terms*, also a positive aspect of video meetings, highlights that video meetings made employees with hearing loss feel more included and empowered, as they experienced the same conditions as their hearing peers. This finding is not explicitly connected to a specific factor within the FUEL. However, it can be interpreted as several factors, including transmission and listener factors, influencing the participants’ positive experiences and making them feel on equal terms with their typically hearing colleagues. However, the equal terms subtheme contradicts studies on in-person meetings, where feelings of exclusion or disadvantage are often reported.^8,47^ Video meetings also required greater discipline and focus from all participants, which some employees with hearing loss found beneficial. Additionally, they could use their hearing devices and technologies without experiencing embarrassment or stigma. This suggests that digital communication can improve the participation of people with hearing loss.

However, those positive aspects of video meetings were counterbalanced by some negative aspects. One of the negative aspects of video meetings was evident in the subtheme of *digital tiredness*. This aligns with previous studies that have reported the challenges and demands of communication in video meetings for people with hearing loss.^36,37^ For instance, Pinsonnault-Skvarenina et al.^28^ found that video meetings require significant technological, structural, and auditory adaptation and can be more energy-consuming and attention-draining than face-to-face communication. Hersh et al.^46^ further found that video conferencing was perceived as ‘overwhelming’ and ‘exhausting’ by disabled participants, highlighting the increased cognitive load and fatigue associated with virtual communication. More broadly, video meetings during COVID-19 were often perceived as particularly tiring, with participants struggling with engagement and communication fatigue in the virtual setting.^43^ This aligns with the overall structure of the FUEL, increasing cognitive demand (e.g., attentional resources), listener dissatisfaction, and motivation. Similarly, some of our participants felt more tired after video meetings than physical ones, attributing this fatigue to transmission factors as referred to in the FUEL.^4^ Such factors include background noise, microphone feedback, and message factors such as difficulty in following conversations. This digital tiredness may differ from the general tiredness that working individuals report in their professional lives.^4^ Another negative subtheme, *mental fatigue*, is consistent with previous studies on employees with hearing loss.^4^ Hersh et al.^46^ also found that participants with disabilities experienced video meetings as overwhelming and exhausting. Importantly, such experiences are not limited to individuals with hearing loss or disabilities. A two-part study conducted in Germany and Israel demonstrated that video conferences were generally more exhausting than other types of meetings, with employees reporting physical and mental fatigue.^48^ Our participants, however, identified specific factors that may increase mental fatigue during video meetings, such as the number of meetings (context factor), the movement of speakers (source factor), and the lack of visual cues (context factor). They reported that this mental fatigue affected their memory and mood after digital meetings. In line with the study by Chauvette et al.,^37^ our participants also reported several challenges in video meetings with many participants, including identifying speakers, managing turn-taking, and staying engaged.

Participants also highlighted the negative aspects of *large meetings*. According to Svinndal et al.,^12^ large meetings were experienced as less engaging and with fewer opportunities to participate fully. The participants also reported difficulty following the conversation when many participants were involved, and the lack of visual cues from too many (or too few) active cameras further complicated participation [see also 31 for the importance of visual cues]. Within the FUEL,^5^ many factors negatively affect participation in large meetings. Context factors complicate following the conversation; too many or too few active cameras affect transmission factors. Large meetings also impact motivation to engage in the listening situation.

*Technological aspects* is another negative subtheme, highlighted by respondents who expressed frustration with poor audio and video quality, background noise, and technical difficulties that made it hard to follow the meetings. These experiences are similar to those reported by Chauvette et al.,^37^ who found that workers with hearing loss often faced challenges in video meetings due to these issues. Our findings further align with a systematic review by de Andrade et al. on remote meetings for software teams, which identified similar challenges, including network connectivity interruptions, audio/video glitches, and software compatibility issues that disrupted communication.^49^ Suggested solutions from our participants included using good microphones, minimising background noise, speaking in turns, and keeping meetings short. These suggestions can reduce the negative impact of transmission factors^5^ on the listening situation. A further negative subtheme was the difficulty of participating in *hybrid meetings*. This setting posed several challenges for employees with hearing loss, such as poor sound quality, lack of visual cues, background noise, and difficulty following turn-taking, especially if they attended digitally. These factors increased the listening effort, leading to exhaustion and frustration.^5^ These challenges align with findings from the systematic review by de Andrade et al.^49^ on remote meetings for software teams, which highlighted broader communication difficulties in online settings. Remote participants often struggled to perceive reactions, gestures, and facial expressions, making it harder to interpret and respond to communication nuances. This reduced attention, engagement, and caused potential breakdowns in shared knowledge and understanding.^49^ While no previous studies have specifically examined the impact of hybrid meetings on employees with hearing loss, it is reasonable to assume that this setting creates more barriers and inequalities than either fully face-to-face or fully online meetings.

The subtheme of *participation* captures employees’ social and professional involvement in video meetings. Some felt less connected and engaged than in face-to-face meetings, where visual and nonverbal cues and social interaction were more accessible. This aligns with research showing that employees with hearing loss often face social barriers like background noise, group conversations, and jokes.^47^ Previous studies have also highlighted the importance of professional participation for employees with hearing loss, as it can increase their self-esteem, job satisfaction, and career opportunities.^4,8^

The *strategies* subtheme of personal responsibility (*my own responsibility*) highlighted how participants informed their communication partners about their hearing loss and requested adjustments. Chauvette et al.^37^ also found that this strategy helped participants gain more patience and understanding [see also 4 for strategies in general working life]. Another strategy identified was *workload distribution*. Participants reported that they avoided scheduling multiple consecutive video meetings within a single day and adjusted their work tasks to align with their available energy levels. This strategy appears specific to communication in video meetings, as previous studies have not reported it in general working life for adults with hearing loss. Participants reduced video meetings’ demands by distributing their workload and improving their recovery. The subtheme of *taking control* also emerged, involving strategies like informing meeting attendees about their hearing loss beforehand. Such strategies are similar to those reported in studies on employees with hearing loss in face-to-face settings.^8^ However, our participants also experienced technological advantages in video meetings, such as controlling the volume and not relying on others to adjust the sound. This strategy may reduce some negative consequences of disclosing one's hearing loss, such as embarrassment, stigma, or reduced self-esteem.^46^

The subtheme of *support* showed the importance of leadership and workplace support for employees with hearing loss and is in line with previous research on working life in general for employees with hearing loss. For example, Svinndal et al.^47^ found that supportive managers and coworkers who actively implement accommodation measures can reduce strain for employees with hearing loss. Our results suggest this is also true for communication in video meetings. Supportive leadership can facilitate adopting and using appropriate technology, such as headsets with microphones, and ensure clear and structured meetings. Supportive coworkers can help their colleagues with hearing loss to follow the conversation and feel included in the social aspects of work. Another support subtheme was the importance of *recovery*, both physical and mental, after engaging in communication in video meetings. This finding aligns with previous research on working life for employees with hearing loss, showing that the need for recovery is greater than that of their normal hearing peers.^8,18,19,50^ However, we also found a positive aspect that differs from physical working life: the experience of more time to recover while working from home.

The FUEL and previous research were used to interpret both positive and negative aspects of video meetings in relation to listening effort. According to the FUEL, listening effort is influenced by the interaction between human factors (e.g., listener's abilities), technological factors (e.g., poor sound quality), and environmental factors (e.g., task and context).^5^ From a more practical stance, FUEL can be used to emphasise the interaction between human, technological, and environmental factors, and can guide practical improvements in the design and implementation of video meeting tools to reduce listening effort and enhance accessibility. For instance, the FUEL highlights the importance of improving audio quality (transmission factor) and ensuring clear visual cues, such as well-lit faces and high-resolution video (context factor). Additionally, simplifying message complexity (message factor) can support individuals who struggle with cognitive or linguistic demands. Moreover, customising meeting platforms to allow for adjustable features, such as personalised sound settings or automatic background noise suppression, could alleviate challenges posed by degraded signals or challenging environments. From a human factor perspective, tools could integrate visual and verbal indicators that enhance focus or provide additional cues for comprehension [see Hadley et al.,^44^ showing the importance of supportive written cues for understanding degraded speech]. Features designed with these principles could help employees with hearing loss and their colleagues better manage attention, retain information, and reduce stress.

### Limitations and future directions

This study has some limitations that should be acknowledged. First, a qualitative approach was used, which is inherently interpretive and shaped by participants’ perspectives and perceptions of their experiences. However, this approach was necessary to explore the various aspects of video meetings for employees with hearing loss. Second, self-selection into the study may have resulted in a sample of more motivated or tech-literate participants, which could limit the diversity of perspectives. Third, the study relied solely on interview data, which may limit the breadth of perspectives captured. Future research could strengthen validity by incorporating additional data sources such as observations, meeting logs, or surveys. Fourth, the focus was on video meetings rather than examining other digital means of communication that have gained popularity in recent years, like Microsoft Teams or Slack. Digital platforms like Teams and Slack offer threaded conversations and real-time messaging with colleagues, presenting different challenges and opportunities for employees with hearing loss. Future research could build on the findings of this study to assess the impact of video meetings on employees using quantitative designs. This could be achieved by developing instruments to measure listening effort in video meetings and conducting experiments to examine specific risk factors associated with listening effort. Furthermore, research could include other digital communication means (e.g., threaded conversations and real-time messaging) and their impact on employees with hearing loss. Finally, future studies could evaluate the benefits of implementing guidelines, such as using high-quality headsets, ensuring good lighting, and encouraging meeting participants to keep their cameras on.

## Conclusion

The current study examined how employees with hearing loss experience video meetings, identifying both the benefits and challenges. Thematic analysis revealed how participation may be influenced and identified strategies that reduce listening effort. The study used the FUEL alongside data-driven thematic analysis to interpret these challenges and inform considerations for workplace improvements. Based on participants’ accounts and these interpretive frameworks, we suggest that technical support and a supportive work environment may be beneficial. High-quality audio, video, captions, and interpreting services may support source factors, while inclusive communication practices may help minimise conversation overlap. Such practices could help reduce listening effort and support broader participation. Employers might consider facilitating transmission by providing quality headsets, allowing remote work, and enforcing turn-taking in hybrid meetings. These approaches could help ease listening effort for employees with hearing loss and foster an inclusive work culture. Future research should investigate the impact of these recommendations by empirically evaluating their effects on well-being and productivity.

## Supplemental Material

sj-docx-1-wor-10.1177_10519815251398498 - Supplemental material for The digital workplace and meeting accessibility: A qualitative study on listening effort in video meetings for employees with hearing loss Supplemental material, sj-docx-1-wor-10.1177_10519815251398498 for The digital workplace and meeting accessibility: A qualitative study on listening effort in video meetings for employees with hearing loss by Erik Marsja, Carine Signoret and Victoria Stenbäck in WORK

## References

[1] Haile LM Kamenov K Briant PS , et al. Hearing loss prevalence and years lived with disability, 1990-2019: findings from the global burden of disease study 2019. Lancet 2021; 397: 996–1009. 33714390 10.1016/S0140-6736(21)00516-X PMC7960691

[2] Tao Y Zhang H Wang D , et al. The prevalence and related factors of hearing loss among adults: a systematic review and meta-analyses. Ann Otol Rhinol Laryngol 2025; 134: 93–101. 39707599 10.1177/00034894241293045

[3] Allen JA Lehmann-Willenbrock N . The key features of workplace meetings: conceptualizing the why, how, and what of meetings at work. Organ Psychol Rev 2023; 13: 355–378.

[4] Granberg S Gustafsson J . Key findings about hearing loss in the working-life: a scoping review from a well-being perspective. Int J Audiol 2021; 60: 60–70. 10.1080/14992027.2021.1881628 33630697

[5] Pichora-Fuller MK Kramer SE Eckert MA , et al. Hearing impairment and cognitive energy: the framework for understanding effortful listening (FUEL). Ear Hear 2016; 37: 5S–27S. 27355771 10.1097/AUD.0000000000000312

[6] Hornsby BWY . The effects of hearing aid use on listening effort and mental fatigue associated with sustained speech processing demands. Ear Hear 2013; 34: 523–534. 23426091 10.1097/AUD.0b013e31828003d8

[7] Kramer SE Kapteyn TS Houtgast T . Occupational performance: comparing normally-hearing and hearing-impaired employees using the Amsterdam checklist for hearing and work. Int J Audiol 2006; 45: 503–512. 17005493 10.1080/14992020600754583

[8] Nachtegaal J Kuik DJ Anema JR , et al. Hearing status, need for recovery after work, and psychosocial work characteristics: results from an internet-based national survey on hearing. Int J Audiol 2009; 48: 684–691. 19863354 10.1080/14992020902962421

[9] Hua H Anderzén-Carlsson A Widén S , et al. Conceptions of working life among employees with mild-moderate aided hearing impairment: a phenomenographic study. Int J Audiol 2015; 54: 873–880. 26140299 10.3109/14992027.2015.1060640

[10] Holman JA Hornsby BWY Bess FH , et al. Can listening-related fatigue influence well-being? Examining associations between hearing loss, fatigue, activity levels and well-being. Int J Audiol 2021; 60: 47–59. 33390065 10.1080/14992027.2020.1853261 PMC8315207

[11] Philips C Jacquemin L Lammers MJ , et al. The impact of unilateral severe-to-profound hearing loss on work productivity and the need for recovery after work. J Occup Environ Med 2025; 67: e478–e485. 10.1097/JOM.0000000000003383 40165459

[12] Svinndal EV Solheim J Rise MB , et al. Hearing loss and work participation: a cross-sectional study in Norway. Int J Audiol 2018; 57: 646–656. 29703092 10.1080/14992027.2018.1464216

[13] Heffernan E Coulson NS Henshaw H , et al. Understanding the psychosocial experiences of adults with mild-moderate hearing loss: an application of leventhal’s self-regulatory model. Int J Audiol 2016; 55: S3–S12. 10.3109/14992027.2015.1117663 PMC5706634 26754550

[14] Lüders D Lopes FC Gonçalves CGDO , et al. Hearing impairment among workers and satisfaction with the use of hearing aids. Work 2022; 71: 661–669. 35253691 10.3233/WOR-205263

[15] Henderson N Hodgson S Mulhern B , et al. A qualitative systematic review of the impact of hearing on quality of life. Qual Life Res 2025; 34: 879–892. 39579270 10.1007/s11136-024-03851-5 PMC11982117

[16] Bess FH Hornsby BW . Commentary: listening can be exhausting—Fatigue in children and adults with hearing loss. Ear Hear 2014; 35: 592–599. 25255399 10.1097/AUD.0000000000000099 PMC5603232

[17] Picou EM Buono GH . Emotional responses to pleasant sounds are related to social disconnectedness and loneliness independent of hearing loss. Trends Hear 2018; 22: 2331216518813243. 30482108 10.1177/2331216518813243 PMC6277757

[18] van der Hoek-Snieders HEM Boymans M Sorgdrager B , et al. Factors influencing the need for recovery in employees with hearing loss: a cross-sectional study of health administrative data. Int Arch Occup Environ Health 2020; 93: 1023–1035. 32507999 10.1007/s00420-020-01556-z PMC7519912

[19] van der Hoek-Snieders HEM Boymans M Dreschler WA . Factors associated with change in the need for recovery and subjective listening effort in employees with hearing loss receiving aural rehabilitation. Int Arch Occup Environ Health 2023; 96: 271–283. 36094620 10.1007/s00420-022-01920-1 PMC9905203

[20] Backenroth-Ohsako GAM Wennberg P Klinteberg BA . Personality and work life: a comparison between hearing-impaired persons and a normal-hearing population. Soc Behav Personal Int J 2003; 31: 191–204.

[21] van Leeuwen LM Goderie T van Wier MF , et al. The longitudinal relationship between speech recognition in noise, need for recovery after work, job demand, and job control over a period of 5 years. Ear Hear 2022; 43: 659. 34619688 10.1097/AUD.0000000000001127

[22] Atcherson SR . Assistive technology for adults with hearing aids. Semin Hear 2022; 43: 079–084. 10.1055/s-0042-1748873 PMC9325080 35903072

[23] Brännström KJ Båsjö S Larsson J , et al. Psychosocial work environment among Swedish audiologists. Int J Audiol 2013; 52: 151–161. 23216266 10.3109/14992027.2012.743045

[24] Yoon CY Lee J Kong TH , et al. Effect of changes in the hearing aid subsidy on the prevalence of hearing loss in South Korea. Front Neurol 2023; 14: 1215494. 37780724 10.3389/fneur.2023.1215494 PMC10536239

[25] Dong S Meros T Seenath S . Workplace accommodation requests: experiences of barriers and facilitators among deaf and hard-of-hearing. Work 2023; 76: 1565–1578. 37355928 10.3233/WOR-220632

[26] Davis MM Devoe M Kansagara D , et al. Did I do as best as the system would let me?’ Healthcare professional views on hospital to home care transitions. J Gen Intern Med 2012; 27: 1649–1656. 22829294 10.1007/s11606-012-2169-3 PMC3509297

[27] Rudner M Lunner T . Cognitive spare capacity and speech communication: a narrative overview. BioMed Res Int 2014; 2014: 869726. 24971355 10.1155/2014/869726 PMC4058272

[28] Stenbäck V Marsja E Hällgren M , et al. The contribution of age, working memory capacity, and inhibitory control on speech recognition in noise in young and older adult listeners. J Speech Lang Hear Res 2021; 64: 4513–4523. 34550765 10.1044/2021_JSLHR-20-00251

[29] Stenbäck V Marsja E Hällgren M , et al. Informational masking and listening effort in speech recognition in noise: the role of working memory capacity and inhibitory control in older adults with and without hearing impairment. J Speech Lang Hear Res 2022; 65: 4417–4428. 36283680 10.1044/2022_JSLHR-21-00674

[30] Koelewijn T Versfeld NJ Kramer SE . Effects of attention on the speech reception threshold and pupil response of people with impaired and normal hearing. Hear Res 2017; 354: 56–63. 28869841 10.1016/j.heares.2017.08.006

[31] Bernstein LE Jordan N Auer ET , et al. Lipreading: a review of its continuing importance for speech recognition with an acquired hearing loss and possibilities for effective training. Am J Audiol 2022; 31: 453–469. 35316072 10.1044/2021_AJA-21-00112 PMC9524756

[32] Nason K Wilbiks J. . Call me maybe: effects of notification modality on visual sustained attention. Multisensory Res 2025; 38: 61–75. 10.1163/22134808-bja10147 40432618

[33] Lavie N . Attention, distraction, and cognitive control under load. Curr Dir Psychol Sci 2010; 19: 143–148.

[34] Lavie N Hirst A De Fockert JW , et al. Load theory of selective attention and cognitive control. J Exp Psychol Gen 2004; 133: 339–354. 15355143 10.1037/0096-3445.133.3.339

[35] Mattys SL Davis MH Bradlow AR , et al. Speech recognition in adverse conditions: a review. Lang Cogn Process 2013; 27: 953–978.

[36] Pinsonnault-Skvarenina A Hotton M Sharp A , et al. Communication during the COVID-19 pandemic: the hearing-impaired perspective. Int J Audiol 2023; 62: 1155–1165. 36129442 10.1080/14992027.2022.2120552

[37] Chauvette L Pinsonnault-Skvarenina A Sharp A , et al. Perceptions of adults with hearing loss about the communication difficulties generated by the COVID-19 preventive measures: a qualitative study. J Speech Lang Hear Res 2023; 66: 5109–5128. 37934877 10.1044/2023_JSLHR-23-00163

[38] Malterud K Siersma VD Guassora AD . Sample size in qualitative interview studies: guided by information power. Qual Health Res 2016; 26: 1753–1760. 26613970 10.1177/1049732315617444

[39] Braun V Clarke V . Conceptual and design thinking for thematic analysis. Qual Psychol 2022; 9: 3.

[40] Fejes A Thornberg R . Handbok i Kvalitativ Analys. 3rd ed. Stockholm: Liber AB, 2019.

[41] Braun V Clarke V . Toward good practice in thematic analysis: avoiding common problems and be (com) ing a knowing researcher. Int J Transgender Health 2023; 24: 1–6. 10.1080/26895269.2022.2129597 PMC9879167 36713144

[42] Lumivero. NVivo, www.lumivero.com (2017).

[43] Johns H Burrows EL Rethnam V , et al. Can you hear me now?” video conference coping strategies and experience during COVID-19 and beyond. Work 2021; 70: 723–732. 34719458 10.3233/WOR-210279

[44] Hadley LV Brimijoin WO Whitmer WM . Speech, movement, and gaze behaviours during dyadic conversation in noise. Sci Rep 2019; 9: 10451. 31320658 10.1038/s41598-019-46416-0 PMC6639257

[45] Signoret C Rudner M . Hearing impairment and perceived clarity of predictable speech. Ear Hear 2019; 40: 1140–1148. 30624251 10.1097/AUD.0000000000000689

[46] Hersh M Leporini B Buzzi M . A comparative study of disabled people’s experiences with the video conferencing tools zoom, MS teams, google meet and skype. Behav Inf Technol 2024; 43: 3777–3796.

[47] Svinndal EV Jensen C Rise MB . Working life trajectories with hearing impairment. Disabil Rehabil 2020; 42: 190–200. 30298745 10.1080/09638288.2018.1495273

[48] Nesher Shoshan H Wehrt W . Understanding “Zoom fatigue”: a mixed-method approach. Appl Psychol 2022; 71: 827–852.

[49] De Andrade ASL Jackson V Prikladnicki R , et al. On meetings involving remote software teams: a systematic literature review. Inf Softw Technol 2024; 175: 107541.

[50] Granberg S Widén S Gustafsson J . How to remain in working life with hearing loss – health factors for a sustainable work situation. Work 2024; 79: 1391–1406. 38875067 10.3233/WOR-230377 PMC11613010

[51] O’Brien BC Harris IB Beckman TJ , et al. Standards for reporting qualitative research: a synthesis of recommendations. Acad Med 2014; 89: 1245–1251. 24979285 10.1097/ACM.0000000000000388

