# Supplementary material for: The digital workplace and meeting accessibility: A qualitative study on listening effort in video meetings for employees with hearing loss
Source: Work. 2025 Dec 8;84(1):197–209. doi: 10.1177/10519815251398498 (PMC13144656; doi:10.1177/10519815251398498)
Supplement: sj-docx-1-wor-10.1177_10519815251398498 - Supplemental material for The digital workplace and meeting accessibility: A qualitative study on listening effort in video meetings for employees with hearing loss [file sj-docx-1-wor-10.1177_10519815251398498.docx]

# Interview Guide (Translated)

Thank you for agreeing to participate in this interview. We are interviewing you to better understand how employees with hearing impairments experience the digitalization of working life, such as working from home and/or working via video meetings. We are interested in your experiences and perspectives. So, there are no right or wrong answers to our questions; we are interested in your experiences. We ask you to turn off the sound on your phone. Participation in this study is voluntary, and your decision is entirely yours. The interview should take about an hour, depending on how much information you want to share. I want to record the interview with your permission because I do not want to miss any of your comments. Since I am recording the interview, I ask you to speak with one person at a time. All responses will be kept confidential. Your anonymized interview responses will only be shared with authorized persons. We will ensure that any information we include in our report does not make it possible to identify you as a participant. You can decline to answer any question or terminate the interview anytime and for any reason. Are there any questions about what I have just explained?

May I turn on the digital recorder?

- How would you describe how hearing impairment affects your work life? What changes have you experienced with working from home or having remote meetings?
- Think of a video meeting or video call that you thought worked well and describe how you experienced that situation, both physically and mentally.
- Think of a video meeting or call that you thought worked poorly and describe how you experienced that situation physically and mentally.
- How do you experience following what is said in a video call or meeting compared to a face-to-face meeting? Is it the same? Different?
- How do you experience participating/collaborating in digital meetings?
- What is your experience regarding the difference between working at a job before the pandemic and meeting digitally?
